# Frequency-bin-encoded entanglement-based quantum key distribution in a reconfigurable frequency-multiplexed network

**DOI:** 10.1038/s41377-024-01696-8

**Published:** 2025-01-16

**Authors:** Anahita Khodadad Kashi, Michael Kues

**Affiliations:** 1 Institute of Photonics, Leibniz University Hannover, 30167 Hannover, Germany; 2 Cluster of Excellence PhoenixD (Photonics, Optics, Engineering – Innovation Across Disciplines), Leibniz University Hannover, 30167 Hannover, Germany

**Keywords:** Applied optics, Quantum optics

## Abstract

Large-scale quantum networks require dynamic and resource-efficient solutions to reduce system complexity with maintained security and performance to support growing number of users over large distances. Current encoding schemes including time-bin, polarization, and orbital angular momentum, suffer from the lack of reconfigurability and thus scalability issues. Here, we demonstrate the first-time implementation of frequency-bin-encoded entanglement-based quantum key distribution and a reconfigurable distribution of entanglement using frequency-bin encoding. Specifically, we demonstrate a novel scalable frequency-bin basis analyzer module that allows for a passive random basis selection as a crucial step in quantum protocols, and importantly equips each user with a single detector rather than four detectors. This minimizes massively the resource overhead, reduces the dark count contribution, vulnerability to detector side-channel attacks, and the detector imbalance, hence providing an enhanced security. Our approach offers an adaptive frequency-multiplexing capability to increase the number of channels without hardware overhead, enabling increased secret key rate and reconfigurable multi-user operations. In perspective, our approach enables dynamic resource-minimized quantum key distribution among multiple users across diverse network topologies, and facilitates scalability to large-scale quantum networks.

## Abstract (web summary)

The first-time demonstration of frequency-bin-encoded entanglement-based quantum key distribution is revealed using a scalable and dynamic quantum network architecture that is resource-efficient and benefits from reconfigurability of telecommunication components.

## Graphical abstract

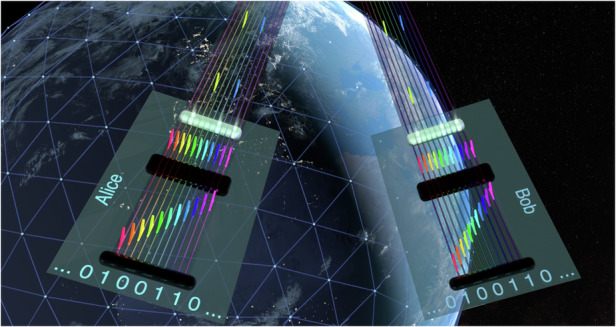

## Introduction

The advent of the quantum internet will introduce a new era of communications, outperforming the classical internet^1^. Central to this new technology is the distributed entanglement-based quantum information processing, which, for example, enables via quantum key distribution (QKD) protocols the establishment of cryptographic keys between distant users^2–7^. Entanglement-based quantum key distribution (EBQKD) protocols offer – compared to prepare-and-measure schemes such as BB84 – an enhanced security against coherent attacks and a greater tolerance to channel loss^8–11^.

Scalability is crucial for large-scale QKD networks, ensuring efficient accommodation of a growing number of users over large distances while upholding security and performance standards^12,13^. However, issues arising from distance limitations, degraded security in face of advanced attacks, resource overhead, and growing hardware complexity/cost emerging from static implementations, hinder a scalable realization of EBQKD^14,15^. In particular, to meet the growing network demands and to accommodate multi-user operations, optimization and stabilization techniques are required to be adapted for polarization^16–19^, time-bin^20–23^, and orbital angular momentum^24–29^ encoding schemes.

The photonic frequency degree-of-freedom has the potential to address the scalability challenge of QKD systems. The inherent multimodal nature of frequency allows for an increased data throughput through parallel transmission of signals at different frequencies. High-dimensional key encryption per photon serves to an intrinsically increased robustness to noise, hence a preserved security^30–34^. Importantly, in contrast to polarization and time-bin encoding, frequency-encoded signals are less susceptible to decoherence induced by environmental factors such as temperature variations and mechanical vibrations. This characteristic supports the development of scalable QKD networks over extended geographical areas without significant loss of quality and coherence. Additionally, the photonic frequency degree-of-freedom could play an interfacing role in hybrid platforms, thanks to the frequency’s convertible nature between different energy-based physical systems^35–37^.

The frequency degree-of-freedom paired with time-bin encoding has been exploited in some QKD protocols, such as prepare-and-measure schemes, measurement-device-independent QKD, twin-field QKD (TFQKD), and has been suggested for the development of quantum network architectures^38–47^. In these implementations, the frequency degree was deployed as part of a hybrid encoding scheme requiring non-scalable bulky interferometric setups with passive or active phase stabilization requirements.

Here, we demonstrate the first-time frequency-bin-encoded implementation of the entanglement-based BBM92 QKD protocol. Additionally, we demonstrate flexible entanglement distribution over long fiber links. By utilizing the frequency-bin encoding approach, we were enabled to develop a novel frequency-bin-basis analyzer module whose employment considerably reduces system complexity and hardware overhead, hence addressing the scalability challenge in large-scale quantum networks. The functionality of this module is based on the deployment of off-the-shelf telecommunication components such as a programmable filter (PF), a frequency mixer (FM) based on electro-optic phase modulation (EOPM), a frequency-to-time mapping (FTM) unit, and a superconducting nanowire single-photon detector (SNSPD) of high timing resolution. With a fine-tuned frequency mixing, our scheme allows for performing passive frequency-bin projection measurements in two mutually unbiased bases (MUB), realizing a random basis choice as a fundamental security condition in QKD protocols^9,10^. Direct frequency-to-time mapping provides each user with simultaneous access to the projection measurement results corresponding to four basis states, using only a single SNSPD. Via an adaptive frequency de/multiplexing functionality we demonstrate the multiplexing of multiple frequency channels for QKD between two users without adding to the hardware overhead. This facilitates enhanced key rates in a single point-to-point QKD with a maintained security level, and enables the scalable expansion of the network to accommodate additional users. Remarkably, we demonstrate that projection states corresponding to multiple quantum channels become as well detectable using the same single detector by each user, hence a maintained system complexity with a growing number of users.

## Results

### Concept and experimental setup of frequency-bin EBQKD

**Fig. 1:**
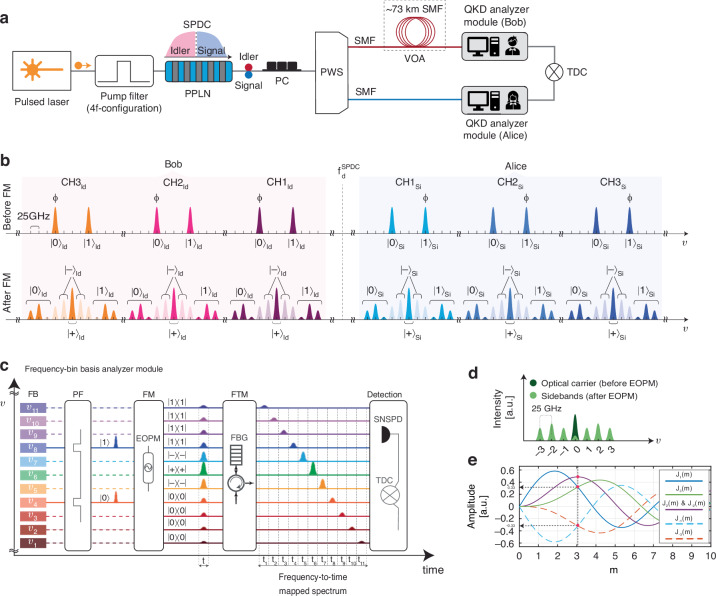
Experimental implementation of frequency-bin-encoded EBQKD. **a** Experimental setup used for the implementation of the BBM92 QKD protocol. **b** Spectral configuration of the BBM92 protocol before and after frequency mixing (FM). The spectrum is created from an SPDC source which spans around the degeneracy frequency ${f}_{d}^{{SPDC}}$ = 193.46 THz. Alice and Bob are allocated with the signal (Si) and idler (Id) spectra, respectively. **c** Frequency-bin basis analyzer module. The projection measurements using the operators of the Z and X basis are displayed in the FM stage. The dotted and solid lines represent the uninvolved and involved spectral positions in each stage, respectively (t: time axis, $\upsilon$: frequency axis). The FTM unit projects the phase-modulated frequency bins from the common temporal mode *t* (realized by the pump pulse) to the distinct temporal modes ranging from ${t}_{1}$ to ${t}_{11}$, corresponding to frequency bins ${\upsilon }_{11}$ to ${\upsilon }_{1}$, enabling a time-resolved detection of the frequency-mixed spectrum. **d** Schematic illustration of EOPM of a single frequency mode, creating up to three sidebands where the ±1st and ∓3rd order sidebands have equal amplitudes. **e** Simulation of sideband generation using Bessel functions: Sideband amplitudes ${J}_{\pm 1:3}\left(m\right)$ versus modulation index m. The dotted vertical black line shows the modulation index m at which ${J}_{\pm 1}\left(m\right)$ and ${J}_{\mp 3}\left(m\right)$ are generated at a maximum identical amplitude. (PPLN: periodically-poled lithium niobate; SPDC: spontaneous parametric down conversion; PC: polarization controller; PWS: programmable wavelength switch; SMF: single mode fiber; VOA: variable optical attenuator; FB: frequency bins; PF: programmable filter; FM: frequency mixing; EOPM: electro optic phase modulation; FTM: frequency-to-time mapping; FBG: fiber Bragg grating; SNSPD: superconducting nanowire single photon detector; TDC. Time-to-digital convertor)

The experimental setup for the frequency-bin-encoded implementation of the BBM92 EBQKD protocol is illustrated in Fig. 1a. A periodically poled lithium niobate (PPLN) waveguide was pumped by a pulsed laser with a repetition rate of 50 MHz and a bandwidth spectrally filtered (via a 4f-configuration optical setup; see Methods and materials) to 200 GHz full-width at half-maximum. The pump photons underwent type-0 spontaneous parametric down conversion (SPDC) process within the PPLN waveguide and split into highly correlated photon pairs distributed symmetrically around the SPDC degeneracy frequency ${{\rm{f}}}_{{\rm{d}}}^{{\rm{SPDC}}}$ = 193.46 THz. The photon-pair source was followed by a central programmable wavelength switch (PWS) separating the propagation path of the higher- (signal) and lower- (idler) energy photons to two users, named Alice and Bob, respectively (see Methods and materials).

The PWS served to define the spectral channels on the signal and idler spectrum (${{\rm{CH}}}_{{\rm{si}}}$ and ${{\rm{CH}}}_{{\rm{id}}}$; see Fig. 1b**;** before FM). Each channel was composed of two frequency bins, labeled $|0\rangle$ and $|1\rangle$. The frequency bins are 20 GHz wide and separated by 100 GHz. The symmetric signal and idler pairs were energy-time correlated with their exact spectral positions determined through correlation measurements. The state amplitude of $|0\rangle$ and $|1\rangle$ were prepared equal to provide a maximally frequency-bin entangled state. To demonstrate scalability, we multiplexed three channels, namely CH1, CH2, and CH3, into the single-mode fiber (SMF) of each user. The channels were separated by 300 GHz on the signal and idler spectrum, respectively. Given the specific definition of the frequency bins in each channel shown in Fig. 1b, the frequency-bin entangled states can be defined as follows:

1 $$\begin{array}{c}{\left|\Psi \right\rangle }^{{\rm{CH}}1}=1/\sqrt{2}\left(\left|0{;}\,t\right\rangle _{{\rm{si}}}^{{\rm{CH}}1}{\left|1{;}\,t\right\rangle }_{{\rm{id}}}^{{\rm{CH}}1}+{\left|1{;}\,t\right\rangle }_{{\rm{si}}}^{{\rm{CH}}1}{\left|0;t\right\rangle }_{{\rm{id}}}^{{\rm{CH}}1}\right),\\ {\left|\Psi \right\rangle }^{{\rm{CH}}2}=1/\sqrt{2}\left(\left|0{;}\,t\right\rangle _{{\rm{si}}}^{{\rm{CH}}2}{\left|1{;}\,t\right\rangle }_{{\rm{id}}}^{{\rm{CH}}2}+{\left|1{;}\,t\right\rangle }_{{\rm{si}}}^{{\rm{CH}}2}{\left|0{;}\,t\right\rangle }_{{\rm{id}}}^{{\rm{CH}}2}\right),\\ {\left|\Psi \right\rangle }^{{\rm{CH}}3}=1/\sqrt{2}\left(\left|0{;}\,t\right\rangle _{{\rm{si}}}^{{\rm{CH}}3}{\left|1;t\right\rangle }_{{\rm{id}}}^{{\rm{CH}}3}+{\left|1{;}\,t\right\rangle }_{{\rm{si}}}^{{\rm{CH}}3}{\left|0{;}\,t\right\rangle }_{{\rm{id}}}^{{\rm{CH}}3}\right)\end{array}$$

where *t* denotes the common temporal mode (defined by the excitation pulse) for the signal and idler frequency bins for all quantum channels. A variable optical attenuator (VOA) was employed to exert incremental optical attenuation corresponding to the loss experienced at different lengths of a SMF placed in Bob’s arm (see Methods and materials), hence creating an asymmetric optical fiber link between the users.

To implement the BBM92 QKD protocol, we developed a novel frequency-bin basis analyzer module (see Fig. 1c), with one of which each user was equipped. This module was composed of a programmable filter (PF), a frequency mixing (FM) unit, a frequency-to-time mapping (FTM) component, a single SNSPD followed by a time-to-digital convertor (TDC).

In general, the BBM92 protocol requires the users to individually perform projection measurements on the received photons by randomly selecting among the two MUB^9,48^, here Z = {$|0\rangle$, $|1\rangle$} and X = {$|+\rangle$, $|-\rangle$} (where $|+\rangle =1/\sqrt{2}\left(|0\rangle +|1\rangle \right)$ and $|-\rangle =1/\sqrt{2}\left(|0\rangle -|1\rangle \right)$). In our approach, the random choice between the Z and X basis and the corresponding projection measurements using the operators {$\left|0\right\rangle \langle 0|,|1\rangle \langle 1|,|+\rangle \langle +|,\left|-\right\rangle \left\langle -\right|$} were realized in the frequency mixing stage.

For simplicity, we explain the operation of the basis analyzer for one given channel. In this module, the PF is configured for each user to let pass the photons of the specified frequency bins with an equal intensity. For clarity, we identify the spectral computational space of a given channel on the signal/idler spectrum with ${\upsilon }_{1}$, …, ${\upsilon }_{11}$ with the input frequency bins $|0\rangle$ and $|1\rangle$ residing in ${\upsilon }_{4}$ and ${\upsilon }_{8}$, respectively, (see Fig. 1c).

Electro-optic phase modulation leads to the generation of sidebands around the original optical carrier at integer multiples of the modulation frequency, here $\Omega$ = 25GHz. In our scenario, the modulation voltage amplitude was fine-tuned (see Methods and materials) to enable the generation of maximum three positive and negative order sidebands (see Fig. 1d). This configuration led to the superposition of $|0\rangle$ and $\left|1\right\rangle$ on the spectral positions ${\upsilon }_{5}$, ${\upsilon }_{6}$ and ${\upsilon }_{7}$.

To experimentally realize the projection measurement onto the $\left|+\right\rangle$ and $\left|-\right\rangle$ states, in these spectral positions, a specific amplitude and phase relationship between the sidebands were required (see Fig. 1b; after FM). The projection onto $\left|+\right\rangle$ was realized in the spectral position ${\upsilon }_{6}$, where the positive and negative second-order sidebands superimpose with an intrinsic equal amplitude and a relative phase difference of 0 (even-order sidebands have an intrinsic phase factor of 0). Projections on $\left|-\right\rangle$ were realized in ${\upsilon }_{5}$ and ${\upsilon }_{7}$, where the first- and third-order sidebands superimpose with a relative phase difference of $\pi$ (negative odd-order sidebands have an intrinsic phase factor of $\pi$). In this realization, it was required to have the first and third-order sidebands ${J}_{\mathrm{1,3}}\left(m\right)$ generated at equal amplitudes. By simulating the sideband generation in electro-optic phase modulation using the Bessel functions (see Fig. 1e), we extracted an optimal modulation RF voltage amplitude (see Methods and materials), where this condition is met, i.e., for a specific modulation index *m* the following equality $|{{\rm{J}}}_{\pm 1}\left(m\right)|=|{{\rm{J}}}_{\mp 3}\left(m\right)|$ is valid. Remarkably, at this modulation index almost maximal amplitude for the second-order sidebands were as well obtained, such that efficient projections onto $|+\rangle$ and $|-\rangle$ were simultaneously feasible. It was verified that the sum of the superposition of first- and third-order sideband amplitudes in ${\upsilon }_{5}$ and ${\upsilon }_{7}$ were with good approximation equal to the superposition of sideband amplitudes in ${\upsilon }_{6}$, realizing equal probability for projections on $|+\rangle$ or $|-\rangle$.

Furthermore, an extent of sideband generation limited to three, renders the spectral region ${\upsilon }_{1}$ to ${\upsilon }_{4}$ and ${\upsilon }_{8}$ to ${\upsilon }_{11}$ devoid of any superpositions. These spectral regions thus represent the projection measurements in the Z basis, realized via the following operators $\left|0\right\rangle \left\langle 0\right|$ and $\left|1\right\rangle \left\langle 1\right|$, respectively (see Fig. 1b**;** after FM). Due to the symmetry in the sideband generation, the probability amplitudes for projection onto $\left|0\right\rangle$ and $\left|1\right\rangle$ states were equal.

Importantly, the sum of the probability amplitudes of the states in Z basis yield nearly identical value to that of the X basis, ensuring that projection measurements in the two MUB occur randomly. In our approach, phase modulation thus projects a single-photon randomly to either the spectral region corresponding to the X basis or to that of the Z basis, hence realizing a stable, passive and random implementation of the projection measurements. The explained concept can be extended to additional frequency channels, hence enabling scalability.

To measure the frequency-mixed single-photon spectrum directly with a single SNSPD (labeled D1 and D2 for Bob and Alice, respectively), the frequency-bins ${\upsilon }_{1}$, …, ${\upsilon }_{11}$ were resolved in time by implementing a FTM technique^49^ using a fiber Bragg grating (FBG) as a dispersive component.

In this experiment, the timing reference between the users is realized using the trigger signal from the common pulsed laser source, captured via the TDC module. By exploiting the pulsed laser signal as a time reference in post processing, the time-resolved spectral components of the single photons became accessible using only a single detector. Given the fine timing jitter of the SNSPD ($\sim$25 ps), the dispersion in the FBG was precisely selected so as to enable time-resolved detection of adjacent 25-GHz frequency bins. Specifically, the FBG possessed by Alice and Bob featured negative and positive second-order dispersion of $\pm$0.0568 ps/nm^2/km, respectively. It is important to note that due to the use of the same pulsed laser as the source of the reference signal for both users and in light of similar timing jitters of both detectors, the accuracy for determining the temporal detection windows associated with the projection basis states for Alice and Bob were identical.

By this detection technique, each user is granted simultaneous access to the projection measurement results in all four basis states {$\left|0\right\rangle ,\left|1\right\rangle ,\left|+\right\rangle ,\left|-\right\rangle$} using only a single detector, hence a considerably reduced resource overhead compared to conventional schemes requiring four detectors^4,48^.

**Fig. 2:**
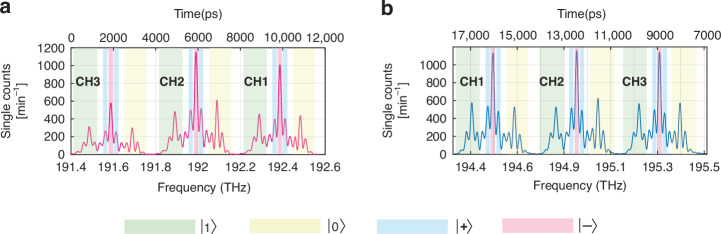
Frequency-to-time mapped spectral profiles of the frequency channels CH1, CH2, and CH3. Frequency-to-time mapped spectral profiles detected by the detectors (**a**) D1 (owned by Bob) and (**b**) D2 (owned by Alice). The colored green, yellow, blue and red spectral regions correspond to the projections onto the states $\left|1\right\rangle$, $\left|0\right\rangle$, $\left|-\right\rangle$, and $\left|+\right\rangle$, respectively

In our approach, the basis analyzer is capable of multiplexing several frequency channels without suffering from hardware overhead. A determining factor for the number of frequency channels that could be resolved is the repetition rate of the pulsed laser. In our case, the 300 GHz spectral distance between the adjacent frequency channels, and the *T* = 20 ns pulse period combined with the dispersion of the FBG, enabled us to demultiplex three frequency channels CH1, CH2, CH3 for each user (see Fig. 2).

### Frequency-bin-encoded BBM92 QKD protocol

With our novel frequency-bin basis analyzer, we implemented a frequency-bin-encoded BBM92 EBQKD protocol (see Methods and materials). The raw key bits in the Z and X basis were obtained from the coincidence events between D1 and D2 and by identifying the spectral regions associated with the following projections $\left|00\right\rangle ,\left|11\right\rangle ,\left|01\right\rangle ,\left|10\right\rangle ,\left|++\right\rangle ,\left|--\right\rangle ,\left|+-\right\rangle ,\left|-+\right\rangle$. The raw coincidence counts in the Z and X basis, ${{\rm{CC}}}_{{\rm{Z}}\left({\rm{X}}\right)}^{{\rm{raw}}}$, consist of the true and accidental counts, i.e., ${{\rm{CC}}}_{{\rm{Z}}\left({\rm{X}}\right)}^{{\rm{True}}}$ and ${{\rm{CC}}}_{{\rm{Z}}\left({\rm{X}}\right)}^{{\rm{Acc}}}$, such that ${{\rm{CC}}}_{{\rm{Z}}\left({\rm{X}}\right)}^{{\rm{raw}}}={{\rm{CC}}}_{{\rm{Z}}\left({\rm{X}}\right)}^{{\rm{Acc}}}+{{\rm{CC}}}_{{\rm{Z}}\left({\rm{X}}\right)}^{{\rm{True}}}$. The true and accidental counts are defined as ${{\rm{CC}}}_{{\rm{Z}}}^{{\rm{Acc}}}={{\rm{CC}}}_{00}+{{\rm{CC}}}_{11}$,${{\rm{CC}}}_{{\rm{X}}}^{{\rm{Acc}}}={{\rm{CC}}}_{+-}+{{\rm{CC}}}_{-+}$, ${{\rm{CC}}}_{{\rm{Z}}}^{{\rm{True}}}={{\rm{CC}}}_{01}+{{\rm{CC}}}_{10}$ and ${{\rm{CC}}}_{{\rm{X}}}^{{\rm{True}}}={{\rm{CC}}}_{++}+{{\rm{CC}}}_{--}$.

The coincidence events were collected over an integration time of *τ* = 1560 s. The coincidence counts averaged over time in each basis are referred to as the raw key rates, i.e., ${{\rm{R}}}_{{\rm{Z}}}={{\rm{CC}}}_{{\rm{Z}}}^{{\rm{raw}}}/\tau$ and ${{\rm{R}}}_{{\rm{X}}}={{\rm{CC}}}_{{\rm{X}}}^{{\rm{raw}}}/\tau$. The ratio between the accidental and the raw coincidence counts in the Z and X basis is termed quantum bit error ratio (QBER) and is denoted by ${{\rm{E}}}_{{\rm{Z}}}={{\rm{CC}}}_{{\rm{Z}}}^{{\rm{Acc}}}/{{\rm{CC}}}_{{\rm{Z}}}^{{\rm{raw}}}$ and ${{\rm{E}}}_{{\rm{X}}}={{\rm{CC}}}_{{\rm{X}}}^{{\rm{Acc}}}/{{\rm{CC}}}_{{\rm{X}}}^{{\rm{raw}}}$, respectively. In Table 1, the experimentally obtained performance metrics for the BBM92 protocol are presented for channels CH1, CH2, and CH3 at different optical attenuations corresponding to different fiber lengths. At 15.6 dB of attenuation, corresponding to $\sim$ 73 km fiber length, the QBER remained for all frequency channels well below the 11% upper bound for a secure QKD protocol^18^, guaranteeing the security of the protocol.

**Table 1:** Performance metrics of the frequency-bin-encoded BBM92 QKD protocol at different optical attenuations corresponding to different lengths of the optical fiber link, prior to error reconciliation and privacy amplification

	Optical attenuation (dB) [corresponding fiber length]					
	0 dB [0 km]	1.64 dB [7 km]	3.71 dB [17 km]	5.73 dB [26 km]	7.98 dB [37 km]	15.6 dB [73 km]
**CH1**						
RCC	25431	18952	12248	7334	4033	652
ACC	1034	759	513	323	158	29
RKR	16.30 ± 0.07	12.14 ± 0.08	7.85 ± 0.07	4.70 ± 0.05	2.58 ± 0.04	0.41 ± 0.01
QBER	(4.12 ± 0.18)%	(4.05 ± 0.15) %	(4.19 ± 0.19) %	(4.40 ± 0.25) %	(3.92 ± 0.32) %	(4.45 ± 0.85) %
**CH2**						
RCC	34603	24571	14464	9513	5504	922
ACC	1797	1198	647	473	273	60
RKR	22.18 ± 0.17	15.75 ± 0.09	9.27 ± 0.07	6.09 ± 0.05	3.52 ± 0.04	0.59 ± 0.01
QBER	(5.20 ± 0.17)%	(4.88 ± 0.14) %	(4.47 ± 0.18) %	(4.97 ± 0.23) %	(4.96 ± 0.32) %	(6.51 ± 0.86) %
**CH3**						
RCC	19485	11941	7322	4490	2937	453
ACC	836	525	315	226	142	28
RKR	12.4904 ± 0.06	7.65 ± 0.07	4.69 ± 0.05	2.88 ± 0.04	1.88 ± 0.03	0.29 ± 0.01
QBER	(4.25 ± 0.21)%	(4.40 ± 0.19) %	(4.30 ± 0.25) %	(5.03 ± 0.34) %	(4.83 ± 0.41) %	(6.18 ± 1.17) %

The results correspond to a measurement integration time of $\tau =$ 1560 s. *RCC* raw coincidence counts, *ACC* accidental coincidence counts, *RKR* raw key rate (per second), *QBER* qubit error ratio

**Fig. 3:**
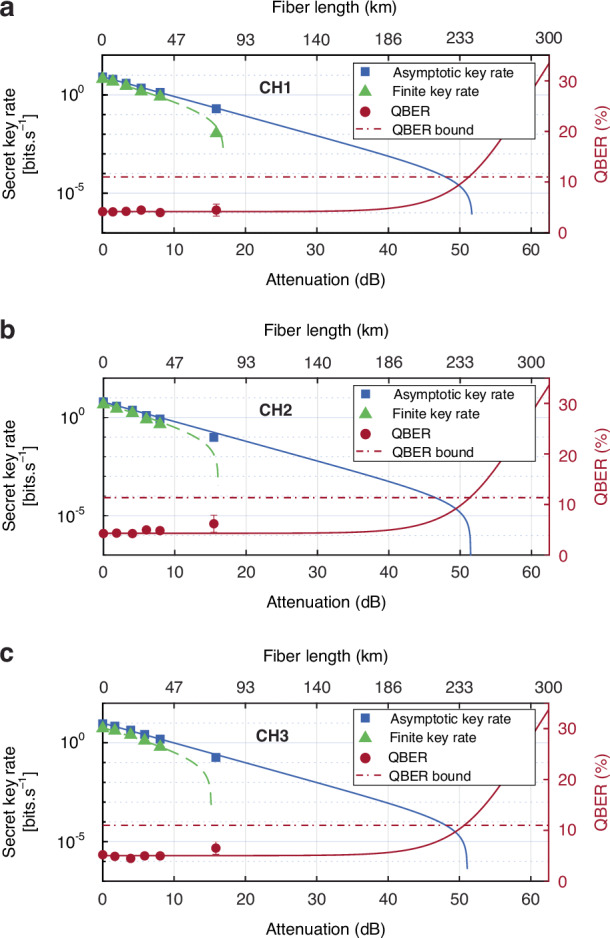
Secret key rate (logarithmic representation) and QBER as function of transmission loss (bottom axis) and the equivalent optical fiber length (top axis). The diagrams correspond to the frequency channels (**a**) CH1, (**b**) CH2, and (**c**) CH3. The experimental results are shown with geometrical colored shapes. The theoretical predictions for asymptotic secret key rate and QBER are indicated with solid blue and red lines, respectively. The theoretical prediction for the finite secret key rate is indicated by the green dotted line. At $\sim$ 15 dB optical attenuation (equivalent to $\sim$ 73 km optical fiber length) the finite secret key rate for CH1 is 0.011/s, whereas for CH2 and CH3 the values are negative and thus no finite key can be extracted. The red dotted horizontal line represents the 11% upper bound of the QBER, below which the security of a protocol with a positive non-zero secret key is guaranteed

We calculated the secret key rate and length for the asymptotic and non-asymptotic (finite) regimes. The asymptotic regime assumes an infinite number of signals to be exchanged between Alice and Bob. Following the Shor-Preskill security proof for an ideal QKD protocol^18^, from the raw key bits we quantified the asymptotic secret key rate ${{\rm{S}}}^{{\rm{A}}}$ (see Methods and materials), shown in Fig. 3. Our theoretical calculations predicted a positive non-zero asymptotic secret key rate for up to 233 km. For a $\sim$ 73 km fiber link, we obtained a maximum asymptotic secret key length ${l}^{A}$ of 310-bits, 283-bits and 149-bits for channels CH1, CH2, and CH3, respectively (see Methods and materials).

In the non-asymptotic regime, a finite number of signals is considered to be exchanged. In practice, the statistical fluctuations introduced by the finite key size effect, combined with failure probabilities in performing error correcting codes, impede the establishment of perfectly secure and correct key bits. Here, we assumed that our protocol is correct and secret up to failure probabilities of ${{\rm{\varepsilon }}}_{{\rm{cor}}}={10}^{-10}$ and ${{\rm{\varepsilon }}}_{\sec }={10}^{-10}$, respectively. (It is important to note that the source-independent nature of EBQKD protocols renders the inefficiencies in the source irrelevant for the secret key analysis). Additionally, we accounted for the asymmetry in the implementation of the protocol arising from unavoidable experimental imperfections. Specifically, an asymmetry in the detection probability in our experimental implementation arises from the different modulation efficiencies in sideband generation, the different quantum channels and in turn the different amounts of loss, the specific selection of detection windows for post-selecting the coincidence events in the Z and X basis states, as well as the slight difference in the intrinsic efficiencies of the detectors. In Table 2, the maximum detection probability mismatch ${\rm{\delta }}$ between the states in the Z basis and the states in the X basis is presented for frequency channels CH1, CH2, and CH3. We accounted for such deviations (from an ideal protocol) by adjusting the amount of privacy amplification (see Methods and materials), using the approach proposed by ref. ^50^. Additionally, we accounted for the detection probability mismatch between the Z and X basis by performing the secret key analysis for each basis independently. The total finite secret key rate ${{\rm{S}}}^{{\rm{F}}}$ in our protocol was thus obtained collectively from the sum of the finite secret key rates in the Z and X basis (see Fig. 3). Our finite secret key analysis provided a positive non-zero secret key rate of up to approximately a fiber length of 73 km. At $\sim$ 15.6 dB optical attenuation (equivalent to $\sim$ 73 km optical fiber length) the finite secret key rate for CH1 has a positive value, whereas for CH2 and CH3 the finite secret key analysis resulted in negative values and thus no secret key could be extracted. This can be explained by the larger overall detection mismatch for channels CH2 and CH3 compared to CH1 (see Table 2), requiring larger amount of raw key bits to be sacrificed for privacy amplification.

**Table 2:** Maximum detection probability mismatch $\delta$ in the Z and X basis for frequency channels CH1, CH2, and CH3

	CH1	CH2	CH3
Z basis	1.72%	6.3%	0.7%
X basis	2.24%	3.41%	3.66%

### Frequency-based entanglement distribution

**Fig. 4:**
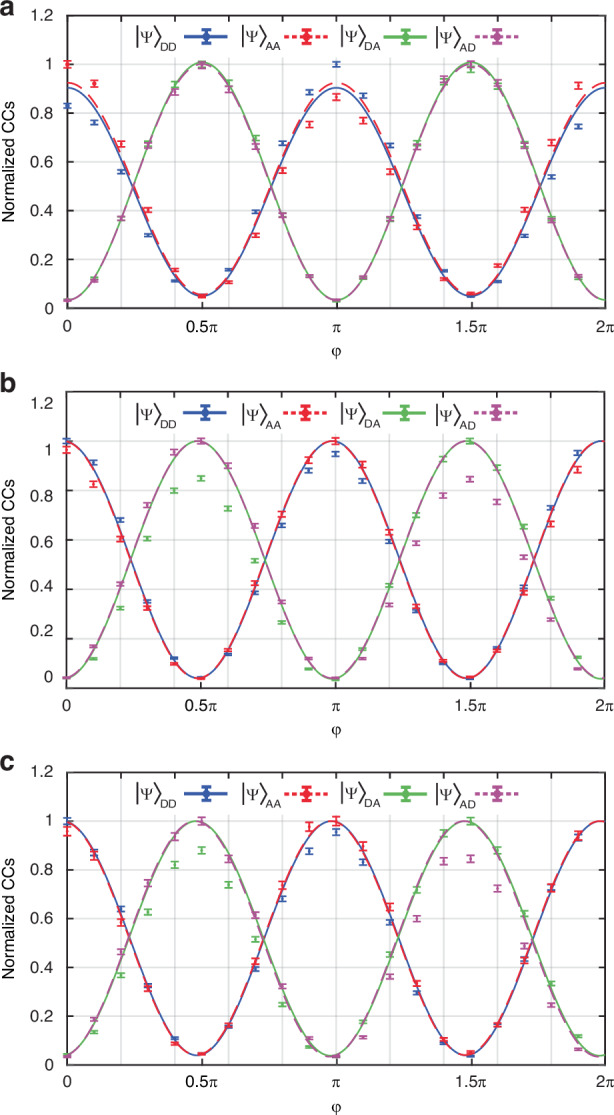
Proof of non-classical correlations. The projection measurements onto the states ${\left|{DD}\right\rangle }_{{Si},{Id}}$, ${\left|{AD}\right\rangle }_{{Si},{Id}}$, ${\left|{AA}\right\rangle }_{{Si},{Id}}$, and ${\left|{DA}\right\rangle }_{{Si},{Id}}$ (shown in different colors) indicate the violation of Bell inequality for the frequency channels (**a**) CH1 (**b**) CH2, and (**c**) CH3. The solid and dotted curves are the theoretical fits and the points are the normalized average coincidence counts measured at different phase values $\varphi$. The error bars are the standard deviation corresponding to an integration time of 1560 s

In our experiment, the SPDC source produces biphoton frequency-bin entangled states. To assess the entanglement quality supported by each channel, we performed Bell-test measurements at zero optical attenuation. In this measurement, Bob and Alice use their programmable filters to impart an identical phase term $\varphi$ on their frequency bins $\left|0\right\rangle$ and $\left|1\right\rangle$, respectively. By sweeping a phase term from $\varphi$ = 0 to 2$\pi$, we performed projection measurements onto the following vector states ${\left|{\rm{\psi }}\right\rangle }_{{\rm{DD}}}=$ ½ (${{\rm{e}}}^{{\rm{i}}{\rm{\varphi }}}{\left|0\right\rangle }_{{\rm{Id}}}+{\left|1\right\rangle }_{{\rm{Id}}}$)$\otimes$(${\left|0\right\rangle }_{{\rm{Si}}}+{{{\rm{e}}}^{{\rm{i}}{\rm{\varphi }}}\left|1\right\rangle }_{{\rm{Si}}}$), ${\left|{\rm{\psi }}\right\rangle }_{{\rm{AA}}}=$ ½ (${{\rm{e}}}^{{\rm{i}}{\rm{\varphi }}}{\left|0\right\rangle }_{{\rm{Id}}}-{\left|1\right\rangle }_{{\rm{Id}}}$)$\otimes$(${\left|0\right\rangle }_{{\rm{Si}}}-{{{\rm{e}}}^{{\rm{i}}{\rm{\varphi }}}\left|1\right\rangle }_{{\rm{Si}}}$), ${\left|{\rm{\psi }}\right\rangle }_{{\rm{DA}}}=$½ (${{\rm{e}}}^{{\rm{i}}{\rm{\varphi }}}{\left|0\right\rangle }_{{\rm{Id}}}+{\left|1\right\rangle }_{{\rm{Id}}}$)$\otimes$(${\left|0\right\rangle }_{{\rm{Si}}}-{{{\rm{e}}}^{{\rm{i}}{\rm{\varphi }}}\left|1\right\rangle }_{{\rm{Si}}}$), and ${\left|{\rm{\psi }}\right\rangle }_{{\rm{AD}}}=$½ (${{\rm{e}}}^{{\rm{i}}{\rm{\varphi }}}{\left|0\right\rangle }_{{\rm{Id}}}-{\left|1\right\rangle }_{{\rm{Id}}}$) $\otimes ({\left|0\right\rangle }_{{\rm{Si}}}+{{{\rm{e}}}^{{\rm{i}}{\rm{\varphi }}}\left|1\right\rangle }_{{\rm{Si}}})$. Here, D and A correspond to measurements on the spectral positions ${\upsilon }_{6}$ as well as ${\upsilon }_{5}$ and ${\upsilon }_{7}$, respectively. The quantum interference corresponding to theses projections are shown in Fig. 4. We calculated the quantum interference visibilities through the following formulation ${\rm{V}}=\left({{\rm{CC}}}_{\max }-{{\rm{CC}}}_{\min }\right)/\left({{\rm{CC}}}_{\max }+{{\rm{CC}}}_{\min }\right)$ where ${{\rm{CC}}}_{\max }$ and ${{\rm{CC}}}_{\min }$ are the maximum and minimum coincidence counts without subtracting the background accidental counts (see Table 3). The obtained visibilities for frequency channels CH1, CH2, and CH3 indicate the violation of Bell inequality (V $>$ 70.71%^31^) by more than $\sim$ 13 standard deviation which certifies the high quality entanglement supported at different spectral regions. The exploited phase difference in odd-order negative sidebands for the basis analyzer is here reflected in a shift of the interference pattern by $\frac{\pi }{2}$ between the projection states ${\left|{\rm{\psi }}\right\rangle }_{{\rm{DD}}}$ and ${\left|{\rm{\psi }}\right\rangle }_{{\rm{AA}}}$ and the projection states ${\left|{\rm{\psi }}\right\rangle }_{{\rm{AD}}}$ and ${\left|{\rm{\psi }}\right\rangle }_{{\rm{DA}}}$.

**Table 3:** Visibilities of the quantum interferences corresponding to the projections vectors ${\left|{DD}\right\rangle }_{{Si},{Id}}$, ${\left|{AD}\right\rangle }_{{Si},{Id}}$, ${\left|{AA}\right\rangle }_{{Si},{Id}}$ and ${\left|{DA}\right\rangle }_{{Si},{Id}}$

	Biphoton projection vector states versus the visibility of quantum interference			
**Channels**	${\left|{\rm{DD}}\right\rangle }_{{\rm{Si}},{\rm{Id}}}$	${\left|{\rm{DA}}\right\rangle }_{{\rm{Si}},{\rm{Id}}}$	${\left|{\rm{AA}}\right\rangle }_{{\rm{Si}},{\rm{Id}}}$	${\left|{\rm{AD}}\right\rangle }_{{\rm{Si}},{\rm{Id}}}$
**CH1**	(89.59 ± 2.23) %	(93.50 ± 2.11) %	(88.66 ± 3.19) %	(93.56 ± 2.25) %
**CH2**	(92.16 ± 1.78) %	(92.68 ± 1.99) %	(92.25 ± 2.45) %	(92.17 ± 2.12) %
**CH3**	(92.43 ± 2.43) %	(92.70 ± 2.71) %	(92.22 ± 3.41) %	(93.45 ± 2.80) %

The visibilities are calculated without background subtraction. The errors correspond to an integration time of 1560 s

**Fig. 5:**
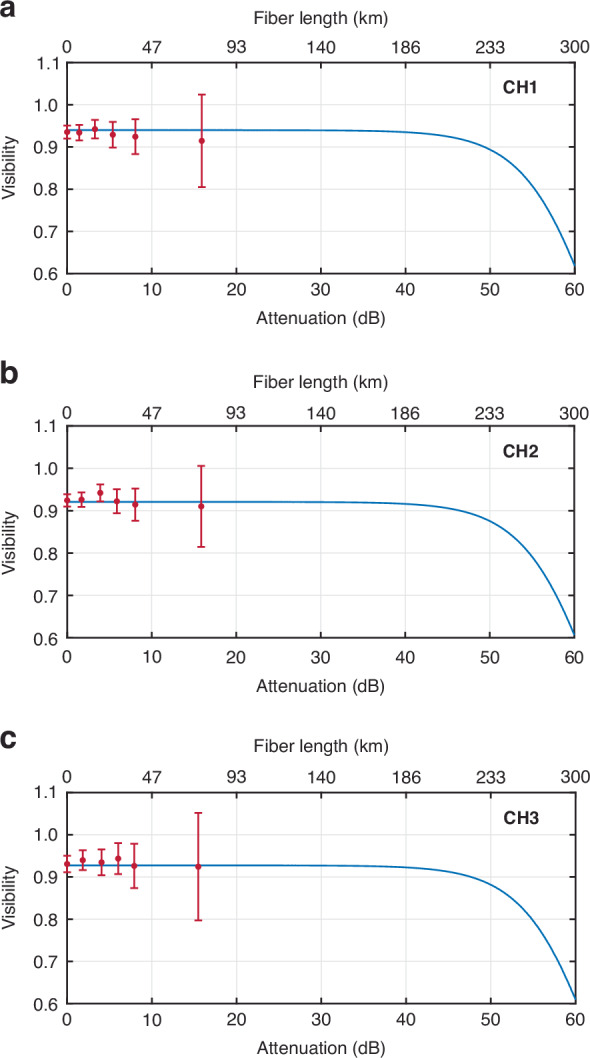
The visibility of quantum interference at different attenuations (bottom axis) corresponding to different optical fiber lengths (top axis). The diagrams correspond to the frequency channels (**a**) CH1, (**b**) CH2, and (**c**) CH3. The experimental data are shown with dots and the solid curves are the theoretical models. The error bars are standard deviations corresponding to a measurement integration time of 1560 s. The increasing standard deviation with increased optical attenuation stems from lower measurement statistics

To assess the capability of our system in preserving entanglement across remote fiber links, we conducted Bell test measurements at varying amounts of optical attenuation. The average visibilities for each frequency channel can be found in Table 4 and Fig.5. We observed that for all frequency channels at $\sim$ 15 dB optical attenuation (equivalent to $\sim$ 73 km fiber link), the average visibility remains above the lower threshold 70.71%, hence the Bell inequality was violated. To obtain the maximum length of the optical fiber link at which high entanglement quality is preserved, we modeled the variation of average visibility as function of channel loss. Our theoretical model adopted from refs. ^4,51,52^ predicts the violation of Bell inequality across all channels up to approximately $\sim$ 55 dB channel loss (equivalent to $\sim$ 257 km fiber link). This underscores the capability of our scheme to enable frequency-insensitive entanglement distribution.

**Table 4:** Average visibility of the quantum interference corresponding to projection vector states ${\left|{DA}\right\rangle }_{{Si},{Id}}$ and ${\left|{AD}\right\rangle }_{{Si},{Id}}$ at different lengths of the optical fiber link for frequency channels CH1, CH2, and CH3

	Average visibility of quantum interference versus different optical attenuations (dB) [corresponding fiber length]					
**Channels**	0dB [0 km]	1.64 dB [7 km]	3.71 dB [17 km]	5.73 dB [26 km]	7.98 dB [37 km]	15.6 dB [73 km]
**CH1**	(93.53 ± 1.54) %	(93.39 ± 1.84) %	(94.22 ± 2.19) %	(92.89 ± 3.04) %	(92.43 ± 4.13) %	(91.44 ± 10.96) %
**CH2**	(92.43 ± 1.45) %	(92.62 ± 1.72) %	(94.20 ± 2.00) %	(92.22 ± 2.84) %	(91.43 ± 3.79) %	(91.02 ± 09.55) %
**CH3**	(93.07 ± 1.95) %	(93.98 ± 2.35) %	(93.46 ± 3.07) %	(94.35 ± 3.66) %	(92.63 ± 5.25) %	(92.43 ± 12.74) %

The standard deviation for each channel corresponds to an integration time of 1560 s

### Network capabilities of frequency-bin-encoded EBQKD

The presented experimental approach can be extended to include multi-user EBQKD network architecture. In such network, the central PWS allows for the specification of an arbitrary number of frequency channels and their dynamic allocation to an arbitrary number of users, hence allowing for different network topologies. According to the graph theory, for a fully-connected network of N users, at least N(N-1)/2 links are required to be established evenly among the users^53–56^. This can be applied to our approach by considering a network with a single entanglement source shared among N users and a PWS that functions as a central service provider multiplexing N-1 frequency channels into the SMF of each user. In such a network, with growing number of users, the network scales linearly since the additional quantum channels are defined within the same source of entanglement.

**Fig. 6:**
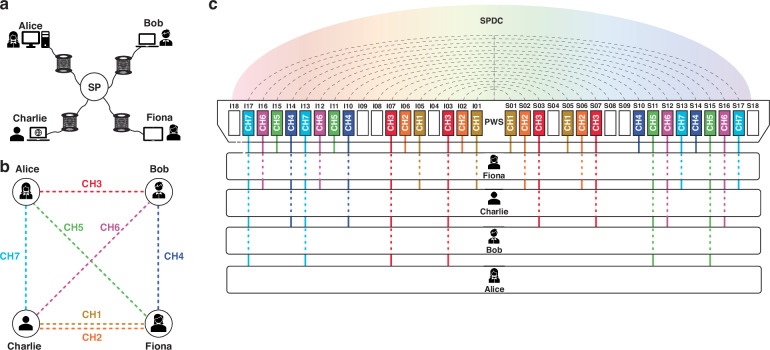
A fully-connected four-user frequency-bin-encoded EBQKD network. Schematic illustration of (**a**) the physical layer, (**b**) the quantum correlation layer, and (**c**) the spectral configuration of the network. The frequency bins belonging to the same channel are presented with the same color and are labeled with an identical channel number ranging from 1 – 7. The pairs of correlated signal and idler frequency bins from the same SPDC process are shown with dotted curves and are numbered from 01 – 18. (SP: Service Provider, PWS: Programmable Wavelength Switch, SPDC: Spontaneous Parametric Down Conversion)

In Fig. 6, a schematic illustration of a fully-connected 4-user EBQKD network is presented. In Fig. 6a, the physical layer of this network is shown, where each user is connected to the service provider via a SMF and owns a personal frequency-bin basis analyzer module. In the quantum correlation layer, at least 6 frequency channels are required to form a fully-connected quantum network. As illustrated in Fig. 6b, each user is linked to the other three users through at least three frequency channels. The specification of the frequency channels and their distribution among the four users to form a fully-connected network is shown in Fig. 6c. As is demonstrated, the channels embrace correlated signal-idler pairs of frequency-bins defined within the broadband spectrum of a single SPDC-based entanglement source. Noteworthy, in this implementation, given the 50 GHz single mode bandwidth and the 25 GHz modulation tone, a frequency spacing of 100 GHz was required to be defined between the frequency bins in each channel. As a result, in a four-user fully-connected EBQKD network, some frequency-bins are left unassigned such as I04, I08 and I09. However, the frequency bins I04 and I08 could also be deployed together as an additional QKD channel and multiplexed to a fifth added user. With this specific spectral configuration for channel allocation, the frequency bin I09 would ultimately be left unassigned as the adjacent frequency bin to I09 needs to be necessarily selected either I13 or I05. Modified RF modulation tones could realize an optimized sideband modulation setting to save on the available frequency bins for allocation of further QKD channels to additional users in the network.

Note that our approach allows for the connection of two users through more than one quantum channel (see Charlie and Fiona in Fig. 6), which increases the key rate while preserving the security as the secret key bits from each channel can be simply added. This is in contrast to increasing the photon flux by applying higher excitation powers aiming at improving the finite secret key rate. In the latter case, the coincidence to accidental ratio will decrease, the entanglement quality degrades, and the QBER will increase accordingly. To create the finite key, more raw key bits will in turn be required. The allocation of more than one quantum channel for finite key extraction can be an alternative solution to increasing the pump power. Note that for the case where two users are connected by more than one quantum channel, the channel in which projection measurement is performed does not necessarily need to be announced in the sifting stage (however, at the cost of increased QBER).

The connection between the users can be dynamically reconfigured enabling to adjust to demand on the key exchange load. Moreover, this fully-connected network architecture can also be reduced to subset topologies to enable QKD between any given two or three users.

## Discussion

We demonstrated a proof-of-concept experimental realization of a frequency-bin encoded BBM92 EBQKD protocol and developed a novel, scalable and resource-efficient frequency-bin basis analyzer module.

Random projection measurements in the Z and X basis were realized simultaneously based on electro-optic phase modulation of the signal and idler photons in each frequency channel. Importantly, as opposed to previous implementations of the BBM92 protocol^57^, the constant phase modulation settings throughout the protocol renders the users needless of any active switching between the different settings to enable the transition between the Z and X basis projection measurements. This is a significant advantage of our approach for that it realizes directly the condition of random choice of basis (required by the BBM92 protocol to ensure security) as well as a speed-up in communications.

Based on the FTM technique, we showcased a frequency demultiplexing capability in time, providing each user with access to the projection states using only a single SNSPD. In contrast to schemes where each user is necessarily equipped with four detectors to detect the four Z and X basis projection states for one quantum channel, the employment of a single detector by each user reduces considerably the resource overhead, minimizes the dark count contribution and the overall vulnerability of the protocol to the detector side-channel attacks. Moreover, the use of a single detector mitigates the imbalance (the asymmetry arising from different amounts of loss encountered in users’ optical paths as well as the detectors’ intrinsic detection efficiency mismatch), required to meet the fair sampling condition to maintain the security level of the protocol. In enabling such characteristics, our approach contributes significantly to reducing the complexity while maintaining the security of the protocol.

The frequency-insensitivity of our approach in preserving high-quality entanglement over long fiber links enables entanglement distribution in quantum networks of metropolitan scale. Noteworthy, with a common transmission mode technique for the RF signal and the optical field, phase stability for the basis state projections required in practical scenarios (see Methods and materials) can be readily achieved. The compatibility of the photonic frequency domain with standard fiber-optic telecommunication infrastructure renders it directly deployable.

The architecture of the presented analyzer module allows for the inclusion of additional frequency channels to the QKD network without any hardware overhead. Remarkably, we showcased the capability of our approach in detecting the projection states corresponding to *N* > 1 quantum channels using the same single detector rather than *N* × 4 detectors. Additionally, for N quantum channels, a single electro-optic phase modulator can be employed rather than N phase modulators, hence providing a simultaneous frequency-mixing and random projection measurements of all channels, in turn addressing the scalability hurdles in large-scale quantum networks.

The FTM technique adopted in our approach provides simultaneous demultiplexing-in-time of the measurement outcomes of all channels to enable their detection with one single detector. However, a tradeoff between the pulsed laser repetition rate and the number of multiplexed frequency channels needs to be considered. The lower the repetition rate of the pulsed laser, the larger the capacity for multiplexing additional frequency channels. The frequency-channel multiplexing can be directly used to increase the key exchange rate in a point-to-point QKD. This is an alternative solution to increasing the pump power, which is unavoidably accompanied by a degraded signal-to-noise ratio, hence detrimental to entanglement fidelity. At the same time, using the same quantum light source and adding further basis analyzers, the frequency-channel multiplexing can be exploited to expand the network for multi-user operations within different network topologies. It is important to note that the efficiency of photon pair generation in SPDC process can differ over its spectral emission range which affects the key exchange rate of different channels. However, the affected key rate of such channels could be compensated by shaping the source’s emission characteristics.

The frequency domain combined with PWS provides an adaptive implementation platform capable of supporting dynamic networking functionalities, in contrast to static wavelength-division-multiplexing components. Specifically, our frequency multiplexing approach can support a dynamic architecture for wavelength allocation to multiple users (hence a further optimized resource-overhead). The basis analyzer directly accepts different frequency channels without a change of system hardware and only by adaptation in measurement operation via a software control. Our approach thus allows for a scalable multi-user QKD network with an adaptive topology, wherein the key rate could be optimized dynamically on-demand with maintained security.

Our proof-of-concept demonstration for frequency-bin EBQKD lays the foundation for further advancements. For example, on-chip integration can be adopted in future steps to reduce loss by an amount of 4–5 dB per module and brightness-enhanced sources could be considered to further improve the key rates. Overall, our solution demonstrates dynamic resource-efficient QKD for multi-user operation with different topologies, thus supporting scalability to future quantum networks.

## Materials And Methods

### Entangled photon pair source, distribution and detection

**Fig. 7:**
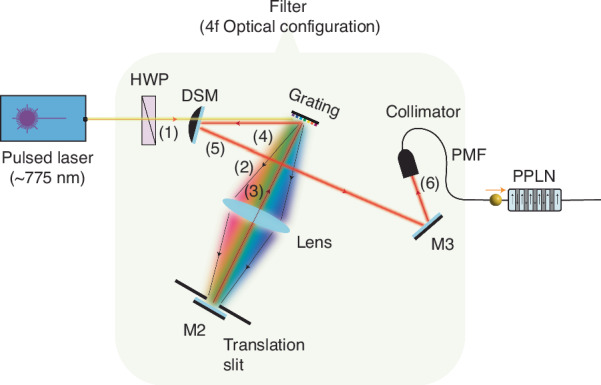
Schematic of a 4f-configuration optical setup employed to spectrally filter the pulsed laser beam to a full-width at half-maximum of 200 GHz centered at 774.82 nm wavelength. The numbers in the figure show the propagation direction of the laser beam. Note that the reflected beam from the Grating denoted by (4) is parallel and underneath the incoming beam denoted by (1). (HWP: half-wave plate; DSM: D-shaped mirror; M: mirror; PMF: polarization maintaining fiber; PPLN: Periodically-poled lithium niobate)

The entangled photon-pair source was composed of a 40 mm-long 5%-MgO-doped PPLN waveguide (*Covesion Ltd*.) excited by 180 μW input power from a mode-locked laser (*C-Fiber 780 Femtosecond Erbium laser by Menlo systems*) with 50 MHz repetition rate and 10 ps pulse duration. The laser beam was spectrally filtered to a full-width at half maximum of 200 GHz via a 4f-configuration optical setup (see Fig. 7) which consisted in a half-wave plate (to adjust the incoming polarization of the laser beam, hence enabling maximum conversion efficiency inside the PPLN), a ruled optical grating, a coated bi-convex lens with a focal length of *F* = 100, and an adjustable mechanical slit to center the output wavelength to the PPLN’s phase matching wavelength of 774.82 nm (386.92 THz). A pulsed-driven type-0 SPDC process within the PPLN waveguide created a broad-band biphoton spectrum over the C-band spectral region. A temperature controller (*Covesion OC3*) was used to maintain the PPLN’s temperature at its phase matching value, 43.4 °C, to enable photon-pair generation with maximum efficiency at the PPLN’s SPDC degeneracy wavelength, 1549.6 nm (193.46 THz).

The frequency channels were defined via the central PWS (*Finisar 4000* *s*; 4.8 dB insertion loss) symmetric with respect to the SPDC degeneracy frequency 193.46 THz (1549.6 nm). The spectral configuration of channels (embracing two frequency bins with 20 GHz bandwidth with 100 GHz frequency spacing) was defined taking into account the single frequency mode bandwidth of 50 GHz full-width at half-maximum, determined in a second-order autocorrelation measurement. The idler and signal frequency bins in each channel were defined at the following center frequencies: ${{\rm{CH}}1}_{{\rm{id}}}$: 192.36, 192.46, ${{\rm{CH}}1}_{{\rm{Si}}}$: 194.46, 194.56, ${{\rm{CH}}2}_{{\rm{id}}}$: 191.96, 192.06, ${{\rm{CH}}2}_{{\rm{Si}}}$:194.86, 194.96, ${{\rm{CH}}3}_{{\rm{id}}}$: 191.56, 191.66, ${{\rm{CH}}3}_{{\rm{Si}}}$: 195.26, 195.36 THz. It is important to note that in this experiment, the operation range of our programmable filter (191.250 to 196.275 THz; specified by the manufacturer; Finisar, 4000S) allowed for the definition of three frequency channels. A spectral filtering device providing a wider selection bandwidth would enable the definition of additional signal and idler frequency channels further apart from the SPDC degeneracy point.

In this experiment, the brightness of our SPDC source was characterized as 5.6 $\times {10}^{5}$ pairs/sec/mW. Under 180 uW input power, the photon pair detection rate for channels CH1, CH2, and CH3 were $\sim$ 695, $\sim$ 796, and $\sim$ 433 pairs/min, corresponding to $\sim$ 3860 pairs/min/mW, $\sim$ 4422 pairs/min/mW, and $\sim$ 2405 pairs/min/mW, respectively. The coincidence to accidental ratio for channels CH1, CH2, and CH3, were obtained to $\sim$ 46, $\sim$ 48, and $\sim$ 50, respectively, signifying the high quality of the time-energy correlated photon pair source.

In this experiment, the FBGs owned by Bob and Alice support a wavelength range from 1528 nm to 1565 nm (equivalent to 191.47 THz to 196.23 THz) and feature approx. maximum insertion loss of 2.23 dB and 2.80 dB, respectively. The SNSPDs feature 100 Hz dark count rate and have approx. a timing jitter of 25 ps. The insertion loss of the components employed in the experimental setup were as follows, PWS: 4.80 dB, PF: 4.8 dB, EOPM_Alice_: 2.98 dB, EOPM_Bob_: 3.91 dB, FBG_Alice_: 2.80 dB, FBG_Bob_: 2.23 dB. In addition, the loss encountered at different lengths of an optical fiber link is exerted via incremental optical attenuation on Bob’s arm (see Table 5**)**.

**Table 5:** Optical attenuation and the corresponding optical fiber length

Optical attenuation (dB)	1.64	3.71	5.73	7.98	15.60
Equivalent optical fiber length (km)	7.65	17.31	26.66	37.14	73.06

In this experiment the detectors were operating in the free running mode. However, in post-processing, detection windows were defined at full-width half maximum, corresponding to four projection states, so as to exclude the contributions from adjacent sidebands. The specific definition of detection windows introduces a photon gating loss which we quantified for Alice and Bob’s channels separately, shown in Table 6. The values in this table indicate that a wider detection window could be considered to obtain higher key rates.

**Table 6:** Gating loss corresponding to the detection windows

	CH1	CH2	CH3
D1 (Bob)	36.34%	31.63%	31.19%
D2 (Alice)	30.35%	30.49%	29.45%

### Coherence stability of frequency-bin states

**Fig. 8:**
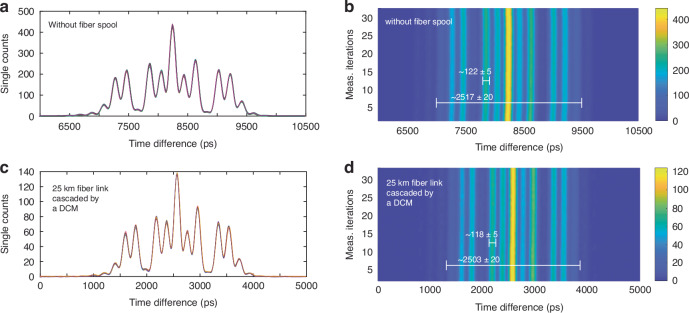
Time-stretched frequency-mixed spectrum of an arbitrary frequency channel. The diagrams correspond to the cases (**a**, **b**) without and (**c**, **d**) with a 25 km optical fiber link cascaded by a dispersion compensating module. The experimental results were obtained for 30 iterations of a measurement with 60 s integration time. (DCM: dispersion compensating module)

The coherence stability of the frequency-bin states was examined under propagation through an optical fiber link of 25 km length cascaded by a dispersion compensating module (DCM). In this experiment, the frequency-mixed single-photon spectrum was measured for two cases of ‘without’ and ‘with’ 25 km fiber link plus a DCM, shown in Fig. 8 a(b) & c(d), respectively. By comparing the results, it is observed that dispersion is compensated over the whole spectrum and that the profile shape of the frequency-mixed spectrum is not distorted. Specifically, the frequency-to-time mapped profiles with 122 ± 5 ps and 2517 ± 20 ps temporal widths (corresponding to $\sim$ 25 GHz and $\sim$ 325 GHz bandwidths, respectively) are maintained after propagation through the fiber link cascaded by a DCM. Moreover, given the 30-min measurement time, the impact of second-order dispersion-induced fluctuations on the spectral profile were measured to be negligible. The measurement results confirm the stability with respect to the reference signal obtained from the pulsed laser source.

In addition, the similarity of the spectral profile before and after transmission through 25 km fiber link signifies that the equivalence between the optical attenuation and the actual optical fiber link is a valid assumption. Noteworthy, for the propagation of single photons through long optical fiber links, non-linear noise, such as e.g. Raman scattering, is not observed.

**Fig. 9:**
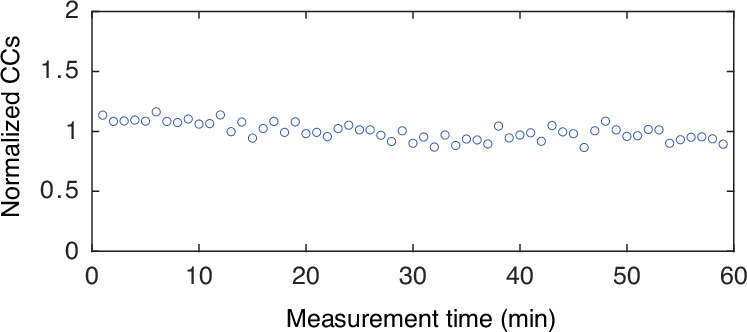
Coincidence counts measured at an arbitrary fixed phase value versus measurement time. The results are normalized to the average 560 coincidence counts per minute

To further assess the coherence stability of the frequency-bin states after propagation through the 25 km-long optical fiber link cascaded by a DCM, we performed a 1-h coincidence measurement from the phase-modulated signal/idler field at a fixed phase value. The normalized coincidence counts versus measurement time are shown in Fig. 9, revealing an almost flat response over time, which demonstrates phase stability for the basis state projection measurements within our thermally controlled laboratory environment. The slight deviation from a perfect phase stable result can be ascribed to slight environmental fluctuations changing the relative photon arrival time with respect to the RF signal.

In practical scenarios, our approach can be adapted to address the larger environmental fluctuations in longer optical fiber lengths. In such cases, by employing a common transmission mode technique using RF signal over optical fiber, the phase instability can be mitigated. In this case, the common transmission mode of the RF tone and the photons’ electric-field allows for their simultaneous accumulation of phase drifts, leading to a stable two-photon interference over time and thus stable projection measurements.

### Electro-optic phase modulation

In electro-optic phase modulation, an arbitrary waveform generator (AWG; Keysight, M8196A) was employed to drive the modulators. The RF signals from channels 1 and 2 of the AWG were precisely adjusted at 415 mV and 240 mV voltage amplitudes to drive the electro-optic phase modulators in possession by Bob and Alice, respectively. The EOMs possessed by Bob and Alice introduced an insertion loss of 3.91 dB and 2.98 dB, respectively.

Noteworthy, the EOMs were required to be phase-stable to maintain the phase relationship for the projections onto the X basis states. In this experiment, the EOMs were intrinsically phase-stable as they were referenced to the same oscillator within the AWG. In practical scenarios where the users reside in their independent laboratories, the transmission of the RF signal over optical fiber can be implemented to achieve phase stability.

### QBER minimization

In this experiment, electro-optic phase modulation was tuned to provide power radiation into sidebands of maximum 3^rd^-order. However, a slight amount of power also radiates into the higher-order sidebands, leading to slight increase in the erroneous projections for the Z basis. Specifically, in post processing, the QBER corresponding to the Z basis was obtained on average between $\sim$ 5.2% to $\sim$ 6.3%, whereas for the X basis, the QBER was obtained roughly within the range from $\sim$ 2.5% to $\sim$ 3.5%. The larger QBER of the Z basis has raised the average QBER to roughly $\sim$ 4% (see Table 1). To minimize the QBER, the detection window for the Z basis states could be defined from the second-order sideband. Alternatively, by tuning the RF signal waveform the sideband creation could be controlled such that the generation of higher order sidebands are better suppressed.

### Polarization stability

In this experiment, the operation of EOPM is polarization-dependent. A polarization-mismatch between the propagating photons and the optical axis of the EOPM would lead to photon loss and in turn a decrease in the detection rate. However, this case does not lead to a rotation of the qubit’s Bloch sphere coordinate system (as it would be the case for polarization encoding), since the qubits in our experiment are defined in the frequency space.

In our experiment, the single detection rates from the signal spectrum was characterized (see Table 7), suggesting that polarization was stable at different steps of the experiment (identified by incremental optical attenuation applied on Bob’s arm).

**Table 7:** Single detection rates (kHz) on D2 (Alice) at different steps of the experiment (identified by incremental optical attenuation applied on Bob’s arm)

Single detection rates (kHz) on D2 (Alice)						
**CH1**	12.22 kHz	12.74 kHz	12.27 kHz	12.32 kHz	12.81 kHz	13.16 kHz
**CH2**	12.52 kHz	13.50 kHz	13.31 kHz	13.33 kHz	13.31 kHz	13.50 kHz
**CH3**	12.15 kHz	11.69 kHz	11.77 kHz	11.63 kHz	11.46 kHz	11.60 kHz

### BBM92 protocol

The BBM92 QKD protocol starts with Alice and Bob each receiving one qubit of the following frequency-bin-encoded entangled pair, represented in the Z and X basis as $\left|{\Phi }_{Z}\right\rangle =1/\sqrt{2}\left({\left|01\right\rangle }_{{\rm{Si}},{\rm{Id}}}+{\left|10\right\rangle }_{{\rm{Si}},{\rm{Id}}}\right)$ and $\left|{\Phi }_{{\rm{X}}}\right\rangle =1/\sqrt{2}\left({\left|++\right\rangle }_{{\rm{Si}},{\rm{Id}}}-{\left|--\right\rangle }_{{\rm{Si}},{\rm{Id}}}\right)$, respectively, with Si and Id denoting Signal and Idler photons, respectively. The users perform independent projection measurements on their qubits based on a random selection among two mutually unbiased measurement basis Z and X. In the sifting stage, the users publicly announce their choices of measurement basis and discard their mismatched selections. In the parameter estimation step, the users reveal a random subset of their signals over the public channel to estimate the statistical error of their protocol. The remaining ${n}_{{\rm{Z}}}$ and ${n}_{{\rm{X}}}$ number of signals are denoted the raw key bits in the Z and X basis, respectively. To compensate for any potential security loss, error correction and privacy amplification steps are implemented and the final secret key string is extracted.

### Theoretical modeling of the QKD performance metrics prior to privacy amplification and error correction

For an EBQKD with a pulsed laser source, the following relationships are valid for the raw key rate per pulse period ${{\rm{Q}}}_{{\rm{Z}}\left({\rm{X}}\right)}$ and the QBER in the Z and X basis denoted by ${{\rm{E}}}_{{\rm{Z}}\left({\rm{X}}\right)}$^51,52^.

2 $${{\rm{Q}}}_{{\rm{Z}}\left({\rm{X}}\right)}=1-\frac{\left(1-{{\rm{D}}}_{{\rm{Z}}\left({\rm{X}}\right)}^{{\rm{A}}}\right)}{{\left(1+{{\rm{\eta }}}_{{\rm{A}}}\frac{{{\rm{\mu }}}_{{\rm{Z}}\left({\rm{X}}\right)}}{2}\right)}^{2}}-\frac{\left(1-{{\rm{D}}}_{{\rm{Z}}\left({\rm{X}}\right)}^{{\rm{B}}}\right)}{{\left(1+{{\rm{\eta }}}_{{\rm{B}}}\frac{{{\rm{\mu }}}_{{\rm{Z}}\left({\rm{X}}\right)}}{2}\right)}^{2}}+\frac{\left(1-{{\rm{D}}}_{{\rm{Z}}\left({\rm{X}}\right)}^{{\rm{A}}}\right)\left(1-{{\rm{D}}}_{{\rm{Z}}\left({\rm{X}}\right)}^{{\rm{B}}}\right)}{{\left(1+{{\rm{\eta }}}_{{\rm{A}}}\frac{{{\rm{\mu }}}_{{\rm{Z}}\left({\rm{X}}\right)}}{2}+{{\rm{\eta }}}_{{\rm{B}}}\frac{{{\rm{\mu }}}_{{\rm{Z}}\left({\rm{X}}\right)}}{2}-{{\rm{\eta }}}_{{\rm{A}}}{{\rm{\eta }}}_{{\rm{B}}}\frac{{{\rm{\mu }}}_{{\rm{Z}}\left({\rm{X}}\right)}}{2}\right)}^{2}}$$

3 $${{\rm{E}}}_{{\rm{Z}}\left({\rm{X}}\right)}={{\rm{e}}}_{0}-\frac{2\left({{\rm{e}}}_{0}-{{\rm{e}}}_{{\rm{Z}}({\rm{X}})}^{{\rm{d}}}\right){{\rm{\eta }}}_{{\rm{A}}}{{\rm{\eta }}}_{{\rm{B}}}\frac{{{\rm{\mu }}}_{{\rm{Z}}\left({\rm{X}}\right)}}{2}\left(1+\frac{{{\rm{\mu }}}_{{\rm{Z}}\left({\rm{X}}\right)}}{2}\right)}{{{\rm{Q}}}_{{\rm{Z}}\left({\rm{X}}\right)}\left(1+{{\rm{\eta }}}_{{\rm{A}}}\frac{{{\rm{\mu }}}_{{\rm{Z}}\left({\rm{X}}\right)}}{2}\right)\left(1+{{\rm{\eta }}}_{{\rm{B}}}\frac{{{\rm{\mu }}}_{{\rm{Z}}\left({\rm{X}}\right)}}{2}\right)\left(1+{{\rm{\eta }}}_{{\rm{A}}}\frac{{{\rm{\mu }}}_{{\rm{Z}}\left({\rm{X}}\right)}}{2}+{{\rm{\eta }}}_{{\rm{B}}}\frac{{{\rm{\mu }}}_{{\rm{Z}}\left({\rm{X}}\right)}}{2}-{{\rm{\eta }}}_{{\rm{A}}}{{\rm{\eta }}}_{{\rm{B}}}\frac{{{\rm{\mu }}}_{{\rm{Z}}\left({\rm{X}}\right)}}{2}\right)}$$

In Eq.(2), ${{\rm{D}}}_{{\rm{Z}}}^{{\rm{A}}}\approx {{\rm{D}}}_{{\rm{Z}}}^{{\rm{B}}}\approx 0.143\times {10}^{-6}$ (sec^−1^) and ${{\rm{D}}}_{{\rm{X}}}^{{\rm{A}}}\approx {{\rm{D}}}_{{\rm{X}}}^{{\rm{B}}}\approx 0.033\times {10}^{-6}$ (sec^−1^) are the dark count rates calculated for 1430 ps and 330 ps coincidence windows of the Z and X basis, respectively, with A and B denoting Alice and Bob. In this implementation, fixed amounts of -10.58 dB and -10.94 dB loss were measured on the optical path of Alice and Bob, respectively. These amounts were resulted from -2.98 dB and -3.91 dB insertion loss from the modulators, -4.8 dB insertion loss from the programmable filters, and 2.80 dB and 2.23 dB loss from the FBGs, for Alice and Bob, respectively. The fixed amounts of loss as well as the intrinsic detector efficiencies ($\sim$ 0.8) were considered in the calculation of the overall transmittance values ${{\rm{\eta }}}_{{\rm{A}}}=$ 0.07 and ${{\rm{\eta }}}_{{\rm{B}}}=$ 0.0644. Given the incremental optical attenuation on Bob’s arm, the overall transmittance for Bob degrades at different optical attenuations, whereas for Alice the amount of loss persists at all lengths of the optical fiber link. In Eq.(2), ${{\rm{e}}}_{0}=$ 0.5 is the error probability, ${{\rm{e}}}_{{\rm{Z}}({\rm{X}})}^{{\rm{d}}}$ is the intrinsic detection error probability arising from the experimental system, and ${{\rm{\mu }}}_{{\rm{Z}}\left({\rm{X}}\right)}$ is the average photon number per pulse period in the Z and X basis. In Table 8, our calculation results for ${{\rm{\mu }}}_{{\rm{Z}}\left({\rm{X}}\right)}$ and ${{\rm{e}}}_{{\rm{Z}}({\rm{X}})}^{{\rm{d}}}$ are presented for CH1, CH2, and CH3.

**Table 8:** Calculation results for the average photon number per pulse period ${\mu }_{Z\left(X\right)}$ and the system detection error probability ${e}_{Z(X)}^{d}$ in the Z and X basis

	CH1	CH2	CH3
${{\rm{e}}}_{{\rm{Z}}}^{{\rm{d}}}$	0.05254	0.06099	0.04969
${{\rm{e}}}_{{\rm{X}}}^{{\rm{d}}}$	0.03008	0.03963	0.03627
${{\rm{\mu }}}_{{\rm{Z}}}$	3.54$\times {10}^{-5}$	5.293$\times {10}^{-5}$	2.91$\times {10}^{-5}$
${{\rm{\mu }}}_{{\rm{X}}}$	3.761$\times {10}^{-5}$	4.895$\times {10}^{-5}$	2.611$\times {10}^{-5}$

To assess the capability of our approach in entanglement distribution over long fiber links, we considered the following equation QBER = $(1-{{\rm{Visibility}}}_{{\rm{ave}}})/2$^4^, relating the QBER and the average visibilities associated with projections onto ${\left|{\rm{DA}}\right\rangle }_{{\rm{Si}},{\rm{Id}}}$ and ${\left|{\rm{AD}}\right\rangle }_{{\rm{Si}},{\rm{Id}}}$ states. With reference to Eq. (2 and 3), the average visibility is thus modeled as function of the channel loss through the following relationship.

4 $${{\rm{Visibility}}}_{{\rm{ave}}}=1-2{e}_{0}+\frac{4\left({{\rm{e}}}_{0}-{{\rm{e}}}_{{\rm{Z}}\left({\rm{X}}\right)}^{{\rm{d}}}\right){{\rm{\eta }}}_{{\rm{A}}}{{\rm{\eta }}}_{{\rm{B}}}\frac{{{\rm{\mu }}}_{{\rm{Z}}\left({\rm{X}}\right)}}{2}\left(1+\frac{{{\rm{\mu }}}_{{\rm{Z}}\left({\rm{X}}\right)}}{2}\right)}{{{\rm{Q}}}_{{\rm{Z}}\left({\rm{X}}\right)}\left(1+{{\rm{\eta }}}_{{\rm{A}}}\frac{{{\rm{\mu }}}_{{\rm{Z}}\left({\rm{X}}\right)}}{2}\right)\left(1+{{\rm{\eta }}}_{{\rm{B}}}\frac{{{\rm{\mu }}}_{{\rm{Z}}\left({\rm{X}}\right)}}{2}\right)\left(1+{{\rm{\eta }}}_{{\rm{A}}}\frac{{{\rm{\mu }}}_{{\rm{Z}}\left({\rm{X}}\right)}}{2}+{{\rm{\eta }}}_{{\rm{B}}}\frac{{{\rm{\mu }}}_{{\rm{Z}}\left({\rm{X}}\right)}}{2}-{{\rm{\eta }}}_{{\rm{A}}}{{\rm{\eta }}}_{{\rm{B}}}\frac{{{\rm{\mu }}}_{{\rm{Z}}\left({\rm{X}}\right)}}{2}\right)}$$

### Secret key rate analysis

We calculate the secret key rate in the asymptotic and finite regime. For the asymptotic secret key analysis, we adopted the following equation ${{\ell}}_{{\rm{Z}}}^{{\rm{A}}}={n}_{{\rm{Z}}}\left(1-{{\rm{H}}}_{2}\left({{\rm{E}}}_{{\rm{X}}}\right)\right)-{{\rm{leak}}}_{{\rm{EC}}}$ to calculate the asymptotic maximum length ${{\ell}}_{Z}^{A}$ of the final secret key in the Z basis^58,59^. Here, ${{\rm{H}}}_{2}$ (e) $=-e{\log }_{2}\left(e\right)-$ (1-e)${\log }_{2}\left(1-e\right)$ denotes the binary entropy function, ${{\rm{leak}}}_{{\rm{EC}}}={n}_{Z}{{\rm{f}}}_{{\rm{e}}}{{\rm{H}}}_{2}\left({{\rm{E}}}_{{\rm{Z}}}\right)$ accounts for the total number of raw key bits used for error correction, and ${{\rm{f}}}_{{\rm{e}}}\ge 1$ is the error correction inefficiency parameter. Here, we consider ${{\rm{f}}}_{{\rm{e}}}=1$ as the Shannon limit for a given error correcting code. ${{\rm{H}}}_{2}\left({{\rm{E}}}_{{\rm{X}}}\right)$ represents the lower bound for the fraction of the raw key bits that are lost during privacy amplification. The same applies for the asymptotic maximum key length in the X basis, ${{\ell}}_{{\rm{X}}}^{{\rm{A}}}$. The total secret key length in the asymptotic regime reads ${{\ell}}^{{\rm{A}}}$ = ${{\ell}}_{{\rm{Z}}}^{{\rm{A}}}$ $+{{\ell}}_{{\rm{X}}}^{{\rm{A}}}$. In the Z basis, we compute the asymptotic secret key rate through the following formulations ${{\rm{S}}}_{{\rm{Z}}}^{{\rm{A}},\left({\sec }^{-1}\right)}={{\rm{R}}}_{{\rm{Z}}}\left(1-{{\rm{H}}}_{2}\left({{\rm{E}}}_{{\rm{X}}}\right)\right)-{\frac{1}{\tau }{\rm{leak}}}_{{\rm{EC}}}$. The same applies to the asymptotic secret key rate in the X basis, ${{\rm{S}}}_{{\rm{X}}}^{{\rm{A}}}$. The total secret key rate in the asymptotic limit is obtained from ${{\rm{S}}}^{{\rm{A}},\left({\sec }^{-1}\right)}={{\rm{S}}}_{{\rm{Z}}}^{{\rm{A}},\left({\sec }^{-1}\right)}+{{\rm{S}}}_{{\rm{X}}}^{{\rm{A}},\left({\sec }^{-1}\right)}$.

In our finite secret key analysis and in the Z basis, we quantify the finite secret key length ${{\ell}}_{{\rm{Z}}}^{{\rm{F}}}$ from the following formulation ${{\ell}}_{Z}^{F}={n}_{{\rm{Z}}}\left(1-{{\rm{H}}}_{2}\left(\frac{{{\rm{E}}}_{{\rm{X}}}+{\rm{\alpha }}}{1-\Delta }\right)\right)-{{\rm{leak}}}_{{\rm{EC}}}-{n}_{{\rm{Z}}}\Delta -\log \frac{2}{{{\rm{\varepsilon }}}_{{\rm{cor}}}{{{\rm{\varepsilon }}}_{\mathrm{sec} }}^{2}}$ which we adopted from^50^. Here, the term $\Delta =1-\frac{1}{{\left(1+\delta \right)}^{2}}$ accounts for the maximum fraction of the key bits in the Z basis removed in privacy amplification to counteract the sources of detection probability mismatch. The parameter ${\rm{\alpha }}$ accounts for the statistical fluctuations associated with the finite key size and is obtained from the following ${\rm{\alpha }}=\sqrt{\left({n}_{{\rm{Z}}}+1\right)\log \left(\frac{1}{{{\rm{\varepsilon }}}_{\sec }}\right)/2{n}_{{\rm{X}}}\left({n}_{{\rm{Z}}}+{n}_{{\rm{X}}}\right)}$^50^. The same applies for the finite secret key length in the X basis, ${{\ell}}_{{\rm{X}}}^{{\rm{F}}}$, and the total finite secret key length reads ${{\ell}}^{{\rm{F}}}={{\ell}}_{{\rm{Z}}}^{{\rm{F}}}+{{\ell}}_{{\rm{X}}}^{{\rm{F}}}$. We compute the finite secret key rate from the following formulations ${{\rm{S}}}_{{\rm{Z}}\left({\rm{X}}\right)}^{{\rm{F}},\left({\sec }^{-1}\right)}={{\ell}}_{{\rm{Z}}\left({\rm{X}}\right)}^{{\rm{F}}}/{\rm{\tau }}$, and the total finite secret key rate of the protocol is calculated from ${{\rm{S}}}^{{\rm{F}},\left({\sec }^{-1}\right)}={{\rm{S}}}_{{\rm{Z}}}^{{\rm{F}},\left({\sec }^{-1}\right)}+{{\rm{S}}}_{{\rm{X}}}^{{\rm{F}},\left({\sec }^{-1}\right)}$. In Table 9, our results of the asymptotic and finite secret key analysis for channels CH1, CH2, and CH3 are presented for different lengths of the optical fiber link.

**Table 9:** Performance metrics of the frequency-bin-encoded BBM92 QKD protocol at different optical attenuations, subsequent to error reconciliation and privacy amplification steps

	Optical attenuation (dB) [corresponding fiber length (km)]					
	0 dB [0 km]	1.64 dB [7 km]	3.71 dB [17 km]	5.73 dB [26 km]	7.98 dB [37 km]	15.6 dB [73 km]
**CH1**						
${{\rm{S}}}^{{\rm{A}}}$ (sec^-1^)	8.28 ± 0.13	6.23 ± 0.11	3.95 ± 0.09	2.24 ± 0.07	1.35 ± 0.05	0.19 ± 0.02
${{\rm{S}}}^{{\rm{F}}}$ (sec^-1^)	6.48	4.75	2.86	1.47	0.81	0.011
${{\rm{S}}}^{{\rm{A}}}$ (N^-1^)	0.50	0.51	0.50	0.47	0.52	0.47
${{\rm{S}}}^{{\rm{F}}}$ (N^-1^)	0.39	0.39	0.36	0.31	0.31	0.026
${{\ell}}^{A}$	12930	9724	6172	3506	2108	310
${{\ell}}^{F}$	10108	7424	4471	2308	1268	17
**CH2**						
${{\rm{S}}}^{{\rm{A}}}$ (sec^-1^)	9.16 ± 0.16	6.95 ± 0.13	4.40 ± 0.10	2.62 ± 0.08	1.51 ± 0.06	0.18 ± 0.02
${{\rm{S}}}^{{\rm{F}}}$ (sec^-1^)	5.30	4.04	2.50	1.26	0.61	−
${{\rm{S}}}^{{\rm{A}}}$ (N^-1^)	0.41	0.44	0.47	0.43	0.43	0.30
${{\rm{S}}}^{{\rm{F}}}$ (N^-1^)	0.24	0.25	0.26	0.20	0.17	−
${{\ell}}^{{\rm{A}}}$	14302	10844	6867	4102	2367	283
${{\ell}}^{{\rm{F}}}$	8271	6303	3901	1979	960	−
**CH3**						
${{\rm{S}}}^{{\rm{A}}}$ (sec^-1^)	6.16 ± 0.12	3.73 ± 0.09	2.34 ± 0.07	1.24 ± 0.05	0.83 ± 0.04	0.09 ± 0.02
${{\rm{S}}}^{{\rm{F}}}$ (sec^-1^)	4.56	2.73	1.67	0.78	0.44	−
${{\rm{S}}}^{{\rm{A}}}$ (N^-1^)	0.49	0.48	0.49	0.43	0.44	0.33
${{\rm{S}}}^{{\rm{F}}}$ (N^-1^)	0.40	0.38	0.36	0.27	0.24	−
${{\ell}}^{{\rm{A}}}$	9609	5820	3659	1944	1298	149
${{\ell}}^{{\rm{F}}}$	7928	4553	2691	1233	726	−

${S}^{A}$ and ${S}^{F}$ denote the asymptotic and finite secret key rate, respectively. *N* refers to the transmitted sifted key. The maximum asymptotic and finite secret key lengths are denoted by ${{\ell}}^{A}$ and ${{\ell}}^{F}$, respectively. The results correspond to an integration time of $\tau =$1560 s

## Data Availability

The data sets used and analyzed during the current study are available from the corresponding author on reasonable request.
